# Defining prevalence of atopic dermatitis in the Avon Longitudinal Study of Parents and Children (ALSPAC)

**DOI:** 10.12688/wellcomeopenres.26317.1

**Published:** 2026-04-30

**Authors:** Morgan Ye, Sinéad M. Langan, Katrina Abuabara

**Affiliations:** 1 Department of Dermatology, University of California San Francisco, San Francisco, USA; 2 London School of Hygiene and Tropical Medicine Faculty of Epidemiology and Population Health, London, England, UK

**Keywords:** ALSPAC, atopic dermatitis, eczema

## Abstract

Longitudinal cohorts with repeated assessments of atopic dermatitis prevalence and severity are rare. Herein, we describe repeated measures from questionnaires and clinical assessments pertaining to dermatitis in the Avon Longitudinal Study of Parents and Children (ALSPAC) birth cohort with the goal of promoting clear and consistent definitions in research. In total, there were 22 time points wherein data were collected relevant to atopic dermatitis (AD). The period prevalence of maternal- or self-reported “rash” in the past 12 months ranged from 11% to 46% across 14 timepoints, with a cumulative prevalence of 80% by age 18. The period prevalence of participant-reported “eczema” in the past 12 months ranged from 13% to 16% across 7 timepoints, with a cumulative prevalence of 29% by age 16. The annual maternal or self-reported period prevalence of “flexural dermatitis,” based on a question similar to large epidemiologic studies, ranged from 14% to 27% across 13 timepoints, with a cumulative prevalence of 57% by age 18. Self-reported point prevalence of flexural dermatitis matched well with estimates from clinical examinations at 6 time points. Additional data on associated infections, medication use, sleep, and school absences are available at limited time points. Based on the quality and consistency of data, we propose creating a new definition of “active” AD: at least 2 reports of flexural dermatitis, up to and including the time point being considered. The annual maternal or self-reported period prevalence of atopic dermatitis based on the 2-report definition ranged from 13–21% across 12 timepoints, with a cumulative prevalence of 41% by age 18, similar to population-based estimates for the UK.

## Introduction

Atopic dermatitis (AD) (also known as atopic eczema or eczema) is the most common chronic inflammatory skin disease worldwide, impacting up to 20% of children and 10% of adults. ^1^ Therefore, understanding its causes, natural history, and potential associations with comorbid conditions is crucial. However, most studies rely on highly selected specialty clinic populations, are cross-sectional, only examine disease course in children, or lack disease activity and severity data. ^2, 3^

The Avon Longitudinal Study of Parents and Children (ALSPAC) offers an opportunity to address many of these literature gaps about long-term outcomes and trajectories in AD. It is a longitudinal birth cohort designed to understand how genetic and environmental factors impact health and development that asks multiple questions about skin symptoms and eczema/AD diagnoses over time. Because AD is a heterogeneous and episodic condition with nonspecific terminology, there exists high potential for misclassification of diagnosis and duration of disease. Herein, we describe repeated measures from questionnaires and clinical assessments pertaining to the definition of AD in ALSPAC with the goal of promoting clear and consistent definitions in research.

## Methods

*Study population:* The Avon Longitudinal Study of Parents and Children is a population-based birth cohort from the UK that recruited pregnant women with expected delivery dates of 1 ^st^ April 1991 to 31 ^st^ December 1992. ALSPAC initially enrolled 14,541 pregnant women, which resulted in 13,988 alive infants at 1 year of age from 14,062 live births. This data note focuses on these index children, which ALSPAC refers to as its G1 cohort. ^4^ The timing for each questionnaire or exam is not exact, but represents an approximate age at data collection. Study details are available on the study website. ^5^ Informed consent was obtained from participants following the recommendations of the ALSPAC Ethics and Law Committee. Ethical approval for the study was obtained from the ALSPAC Ethics and Law Committee and the Local Research Ethics Committees. The completion of a questionnaire, either on paper or online, was considered to be written consent from participants to use their data for research purposes. For the majority of tests undertaken during face-to-face visits, verbal consent was obtained from participants (both parents and children as appropriate) prior to the start of any data collection. However, some tests required the completion of a written consent form. A full list of all ALSPAC ethical approvals can be found here: http://www.bristol.ac.uk/alspac/researchers/research-ethics/. This study was exempt from institutional review board approval by the University of California, San Francisco because investigators did not have access to identifiable data. All analyses were performed using STATA SE 18.0 software (Stata Corp, Texas).

*Available data relating to rash/eczema/atopic eczema/atopic dermatitis:* In total, there were 22 time points wherein data were collected relevant to this topic.

The primary caretaker (most often the mother) or participants were asked standardized questions about flexural dermatitis, eczema, rash, and symptom severity and infections at multiple time points between age 4 weeks to 18 years. Mothers reported on their children’s symptoms at ages 4 weeks, 6 months, and 1, 2, 3, 4, 5, 6, 7, 8, 10, 11, 13, and 16 years. Participants reported on their own symptoms related to flexural dermatitis on questionnaires at ages 16 and 18 years. Some participants also underwent clinic assessments for eczema and flexural dermatitis during a sub-study entitled ‘Child in Focus’ at ages 4–5 years, and at ages 7, 9, 10, 11, 12, and 13 years. Supplemental Table 1 ^6^ provides an overview of these relevant ALSPAC variables at each timepoint.

*Flexural dermatitis:* Participants or their caretakers were asked about flexural dermatitis at age 4 weeks, 6 months, and 1, 2, 3, 4, 5, 6, 8, 10, 11, 13, 16, and 18 years with the question, “Have you/Has your child had itchy, dry skin rash in the joints and creases of the body (e.g. behind the knees, elbows, under the arms) in the past year”. At the following ages, a time frame other than a year was specified: at ages 4 weeks and 6 months, they were asked about ever having flexural dermatitis since birth, and at 4 and 5 years, they were asked about having flexural dermatitis since age 3 and having flexural dermatitis in the past 15 months, respectively.

These questions, except at age 18 years, were followed by a severity question to rank their/their child’s flexural dermatitis as “no problem, mild, very bad, or quite bad;” and a question about history of infection: “Did the rash ever become sore and oozy,” at ages 2, 3, 4, 5, 6, 8, 10, 11, 13, and 16 years.

At these same times points, except for ages 16 and 18 years, participants were also asked a question about whether the child was currently experiencing symptoms (i.e. whether they had the rash “now”). History of rash clearance was assessed at ages 10, 11, 13, 16, and 18 years with a question that asked about whether the rash “cleared completely” over the past 12 months. At ages 10 and 13, they were additionally asked whether they *ever* had flexural dermatitis.

Participants were also examined at clinical centers at ages 7, 9, 10, 11, 12, and 13 years, and observers noted the presence of any flexural dermatitis >1 cm in diameter in *each* of the following areas: around the eyes, the sides or front of the neck, in front of the elbows, behind the knees, or in front of the ankles.

*Rash:* At ages 6 months and 1, 2, 3, 6, 7, 8, 10, 13 (asked two times), 16, and 18 years, participants were asked whether they or their child has had rash in the past 12 months. At ages 4 and 5 years, they were asked about ever getting a rash since age 3 and having a rash in the past 15 months, respectively. At age 16 years, mothers additionally reported whether their child ever had a rash that came and went for at least 6 months. At ages 2, 3, 6, 8, 11, and 16 years, separate questions about itchy dry rash on the hands and feet in the past year were asked. At ages 4 and 5 years, they were asked about itchy dry rash on the hands and feet since age 3 and having itchy dry rash on the hands and feet in the past 15 months, respectively. At age 16, mothers were additionally asked about these rashes on the face and trunk area. At 4 weeks, 6 months, and 18 months, mothers reported whether their babies had convexity dermatitis (i.e. “rash on the face, forearm, or shins”) since birth, currently, or in the past year. If they responded yes, they also reported the severity of the rash, where responses options included “no problem, mild, very bad, or quite bad.”

*Eczema:* At ages 6, 7, 8, 10, 13, 14 (rounded up from 13 years and 10 months) 16, and 18 years, participants were asked about having eczema. At all ages except for age 18, they were asked, “Has you/your child had eczema in the past year?” At ages 10, 13, 16, and 18 years, they were asked whether they/their child ever had eczema, and at ages 10 and 13, they were additionally asked whether a doctor has ever said their child has had eczema. At ages 4 and 5 years, a sub-study, Child in Focus, examined participants for eczema in clinic and the total number of eczema patches were recorded.

*Other variables related to dermatitis:* Mothers reported the number of days off school because of eczema or itchy rash in the past year at ages 7, 8, 10, 11, and 13 years. At age 7 and 8, this question also included either asthma or hay fever. They were also asked about how sleep is affected using the question, “Frequency child has been kept awake by itchy rash” at ages 6, 8, 10, 11, and 13, and 16. At ages 10, 11, and 13 years, questions about medications were asked, including whether they have taken medicine, pills, drops, or ointments for eczema and the frequency of medication in the past year with responses categorized as “regularly, few days, odd occasions, and once or twice.”

*Proposed definition of active atopic dermatitis*: The questions about flexural dermatitis were most consistent with the widely used and validated International Study of Asthma and Allergies in Childhood (ISAAC) questions and repeated at the most timepoints. Given that the definition of atopic dermatitis requires an episodic rash persisting for at least 6 months, prior studies using the ALSPAC database had required at least two positive responses to flexural dermatitis, ^7^ and the cumulative prevalence of flexural dermatitis was higher than most estimates of atopic dermatitis in the UK, we propose a new definition of atopic dermatitis (AD) for use in ALSPAC:

At least 2 reports of flexural dermatitis, up to and including the time point being considered. On the first report of flexural dermatitis, individuals were categorized as being indeterminate/maybe for atopic dermatitis. Individuals were classified as having inactive AD if they met the definition of *active* atopic dermatitis previously but responded negatively at the time point being considered. This results in a four-category AD definition – never, inactive, maybe, and definite AD. Data at 4 weeks was not used because of high rates of seborrheic dermatitis misdiagnosis at that age. At ages 6 months and 1 year, AD was defined by those who answered yes to flexural or convexity dermatitis. ^8, 9^

AD severity was based on a repeated question asking participants/mothers to rank their/their child’s flexural dermatitis and was only calculated for those who were in the definite AD category. We categorized severity categories as no problem, mild, moderate (quite bad), and severe (very bad). At ages 6 months and 1 year, AD severity was based on the highest severity reported for either flexural or convexity dermatitis.

Point prevalence of self-reported AD was calculated based on the report of current flexural dermatitis. Point prevalence of AD from standardized clinical exam was based on clinical data from a subset of individuals at 8 time points, as described above.

## Results

Rates of participant-reported skin symptoms are shown in Table 1 and Supplemental Table 2 ^6^ detailed below. Missing data for the 2-point definition of atopic dermatitis and the flexural dermatitis variables are reported in Supplemental Table 3. ^6^

**Table 1.** Prevalence and severity of atopic dermatitis based on 2 reports of flexural dermatitis.

	Approximate age					
	6 months	18 months	30 months (2y 6 m)	42 months (3y 6 m)	57 months (4y 9 m)	69 months (5y 9 m)
***Period prevalence of participant-reported flexural dermatitis***	3644 (32.0)	3662 (33.1)	2703 (26.6)	2261 (22.7)	2259 (24.3)	1841 (21.6)
Caregiver- reported severity						
No problem	575 (16.7)	530 (14.5)	298 (11.0)	245 (10.9)	251 (11.0)	213 (11.5)
Mild	1713 (49.6)	1973 (53.9)	1440 (53.4)	1265 (56.1)	1385 (60.7)	1139 (61.3)
Quite bad	907 (26.3)	954 (26.1)	756 (28.0)	582 (25.8)	528 (23.1)	414 (22.3)
Very bad	256 (7.4)	201 (5.5)	205 (7.6)	162 (7.2)	119 (5.2)	92 (5.0)
***Point prevalence of participant-reported flexural dermatitis***	1430 (12.5)	1836 (16.6)	1434 (14.1)	1143 (11.5)	1012 (10.9)	819 (9.6)
***Point prevalence of dermatitis from standardized clinical exam*** ^***b***^	- (−)	- (−)	- (−)	- (−)	140 (13.9)	119 (12.2)
***Proposed definition of atopic dermatitis*** ^***c***^						
Never	- (−)	5853 (52.9)	4740 (46.6)	4346 (43.6)	3813 (40.9)	3314 (38.9)
Inactive	- (−)	- (−)	620 (6.1)	1153 (11.6)	1288 (13.8)	1575 (18.5)
Maybe	- (−)	3473 (31.4)	2,812 (27.6)	2545 (25.6)	2255 (24.2)	1958 (23.0)
Definite	- (−)	1740 (15.7)	2005 (19.7)	1914 (19.2)	1960 (21.0)	1682 (19.7)
No problem	- (−)	202 (1.8)	191 (1.9)	181 (1.8)	191 (2.1)	177 (2.1)
Mild	- (−)	887 (8.0)	1037 (10.2)	1064 (10.7)	1170 (12.6)	1038 (12.2)
Moderate	- (−)	518 (4.7)	602 (5.9)	513 (5.2)	484 (5.2)	376 (4.4)
Severe	- (−)	129 (1.2)	170 (1.7)	149 (1.5)	114 (1.2)	87 (1.0)

	81 months (6y 9 m)	103 months (8y 7 m)	128 months (10y 8 m)	140 months (11y 8 m)	166 months (13y 10 m)	198 months (16y 6 m) ^a^	216 months (18y) ^a^
***Period prevalence of participant-reported flexural dermatitis***	1640 (19.5)	1668 (21.2)	1320 (17.8)	1385 (19.7)	915 (13.7)	828 (17.3)	499 (15.7)
Caregiver reported severity							
No problem	177 (10.8)	223 (13.2)	126 (9.5)	152 (11.0)	77 (8.4)	103 (12.0)	- (−)
Mild	1041 (63.7)	1052 (62.4)	911 (68.3)	936 (67.6)	607 (66.2)	423 (49.2)	- (−)
Quite bad	330 (20.2)	331 (19.6)	260 (19.5)	243 (17.6)	196 (21.4)	255 (29.7)	- (−)
Very bad	86 (5.3)	79 (4.7)	37 (2.8)	54 (3.9)	37 (4.0)	78 (9.1)	- (−)
***Point prevalence of participant-reported flexural dermatitis***	689 (8.2)	611 (7.8)	500 (7.8)	487 (6.9)	343 (6.1)	- (−)	- (−)
***Point prevalence of dermatitis from standardized clinical exam*** ^***b***^	623 (8.1)	570 (7.8)	547 (7.7)	416 (6.2)	303 (5.2)	- (−)	- (−)
***Proposed definition of atopic dermatitis*** ^***c***^							
Never	3196 (38.0)	2856 (36.3)	2590 (35.0)	2348 (33.4)	2216 (33.1)	1439 (30.0)	934 (29.5)
Inactive	1831 (21.8)	1821 (23.1)	2019 (27.3)	1937 (27.6)	2209 (33.0)	1636 (34.1)	1157 (36.5)
Maybe	1851 (22.0)	1724 (21.9)	1572 (21.2)	1463 (20.8)	1404 (21.0)	1028 (21.4)	620 (19.6)
Definite	1525 (18.1)	1474 (18.7)	1219 (16.5)	1281 (18.2)	861 (12.9)	697 (14.5)	461 (14.5)
No problem	161 (1.9)	173 (2.2)	107 (1.5)	137 (2.0)	63 (0.9)	61 (1.3)	- (−)
Mild	967 (11.5)	928 (11.8)	830 (11.2)	862 (12.3)	567 (8.5)	354 (7.4)	- (−)
Moderate	311 (3.7)	299 (3.8)	243 (3.3)	229 (3.3)	191 (2.9)	213 (4.4)	- (−)
Severe	81 (1.0)	72 (0.9)	34 (0.5)	50 (0.7)	37 (0.6)	65 (1.4)	- (−)

^a^ Child-reported

^b^ Timepoints are not exact; used timepoint closest to exam timepoint

^c^ Based on our 2-point definition

**Figure 1.**
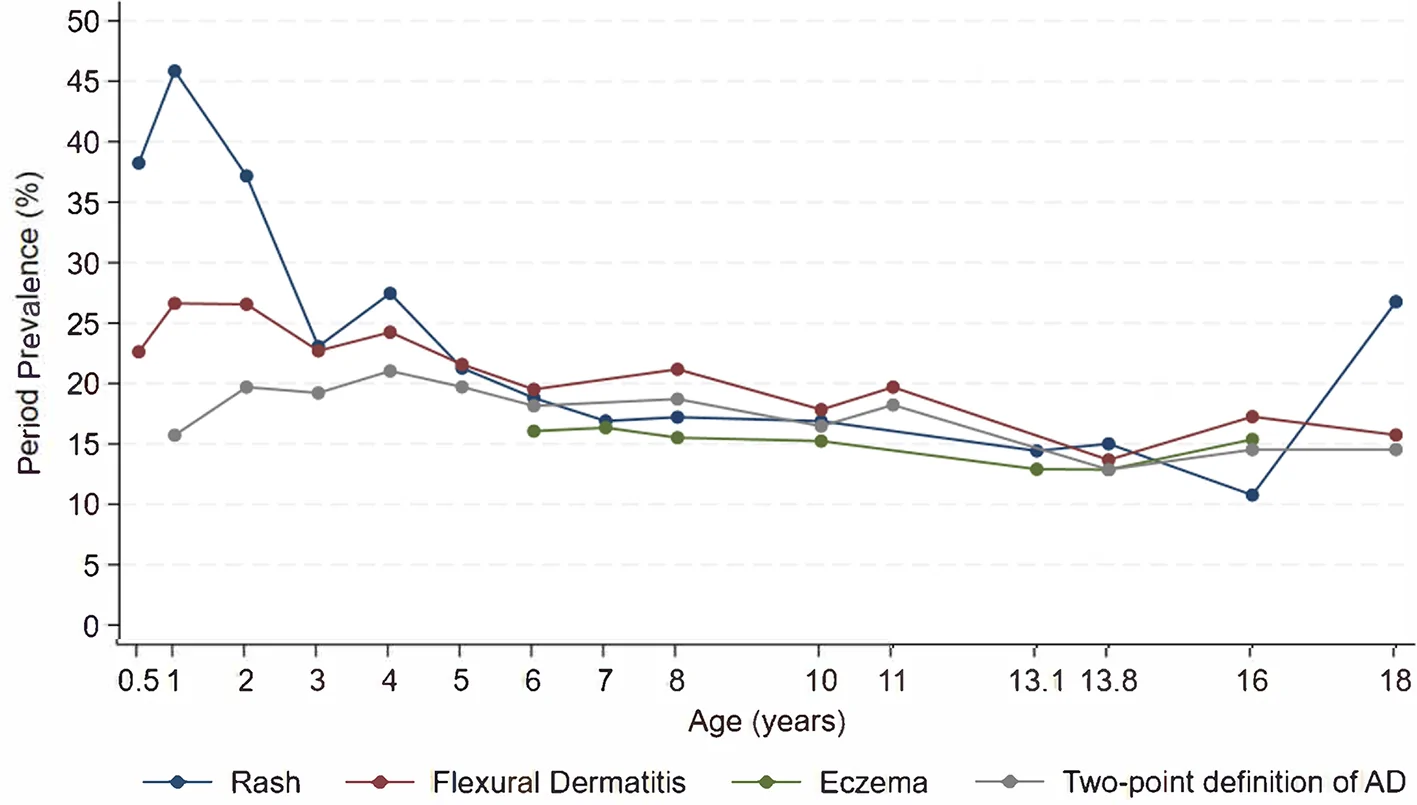
Period prevalence of participant-reported skin symptoms.

*Rash:* The period prevalence of maternal- or self-reported “rash” in the past 12 months ranged from 11% to 46% across 14 timepoints, with a cumulative prevalence of 80% by age 18 ( Figure 1). The number of non-missing responses ranged from 11,428 at 6 months to 3,171 at 18 years. The prevalence of rash on the hands and feet across 8 timepoints ranged from 3% to 7%, with 10,191 non-missing responses at 2 years to 4,796 at 16 years. The period prevalence of convexity dermatitis since birth or in the past 12 months ranged from 14% to 18% from 4 weeks to 18 months, with severity ranging from 11–20% for no problem, 46–53% for mild, 21–32% for quite bad, and 5–7% for very bad dermatitis. The point prevalence for current convexity dermatitis from 4 weeks to 18 months ranged from 7% to 9%. For both point and period prevalence of convexity dermatitis, the number of non-missing responses ranged from 11,016 to 12,071 (Supplemental Table 2 ^6^).

*Eczema*: The period prevalence of eczema in the past 12 months ranged from 13% to 16% across 7 timepoints ( Figure 1), with a cumulative prevalence of 29% by age 16 Figure 1. The number of non-missing responses ranged from 8,476 at 6 years to 4,761 at 16 years. For clinical data, at ages 4 and 5 years, there were 1,009 and 972 non-missing responses, with prevalence of 13.9% and 12.2% and average number of eczema patches of 0.7 (standard deviation (SD) 2.5) and 0.6 (SD 2.3), respectively (Supplemental Table 2). ^6^

**Figure 2.**
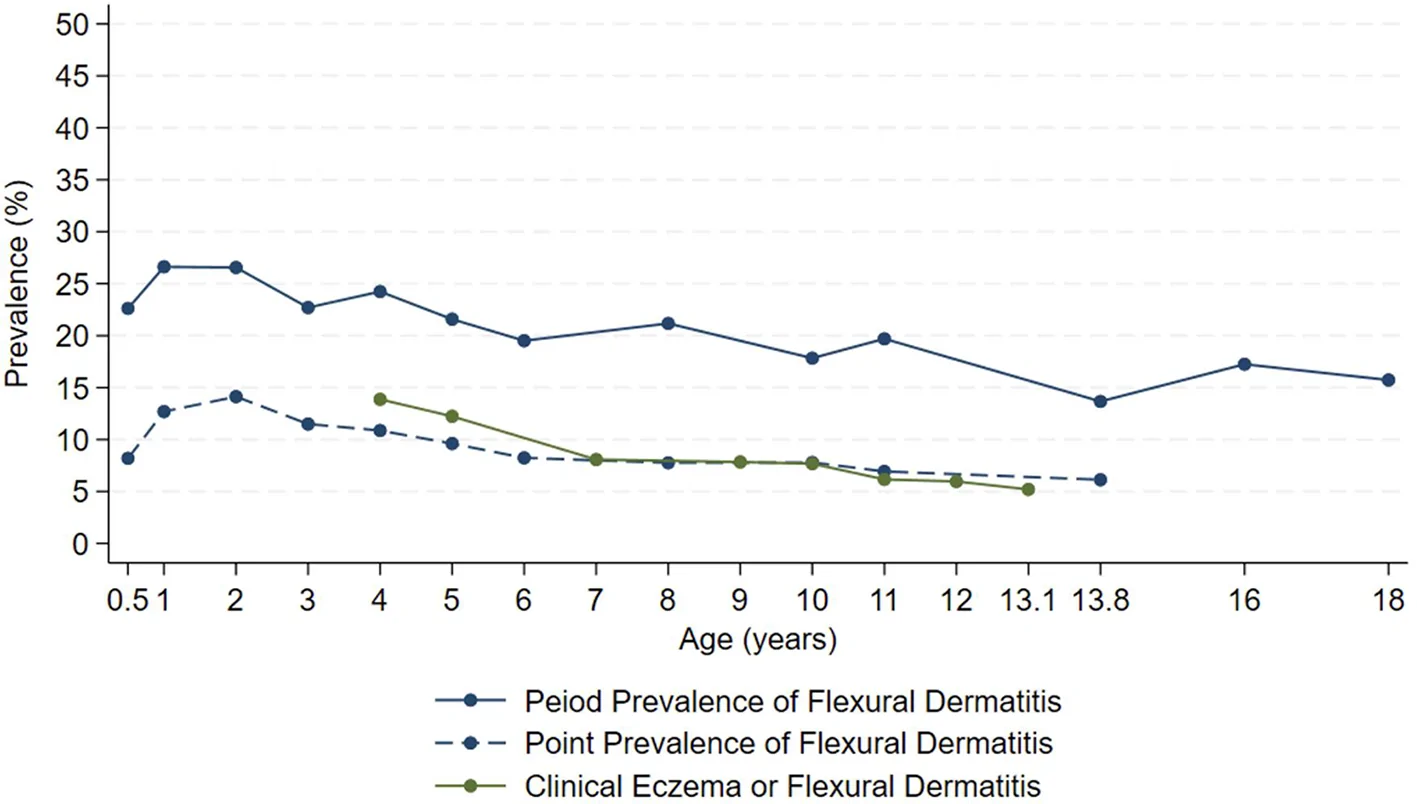
Period and point prevalence of self-reported flexural dermatitis and prevalence of eczema or dermatitis at clinical examination.

*Flexural dermatitis:* The annual maternal or self-reported period prevalence of flexural dermatitis ranged from 14% to 27% across 13 timepoints ( Figure 1), with a cumulative prevalence of 57% by age 18 Figure 1. The number of non-missing responses ranged from 12,177 at 4 weeks to 3,171 at 18 years. Severity ranged from 8–20% reporting the dermatitis was “no problem”, 49–68% for “mild”, 18–30% for “quite bad”, and 3–9% for “very bad”. The percentage of missing data for period prevalence and severity for each timepoint ranged from 1–2%. Point prevalence of participant-reported flexural dermatitis ranged from 6–14%, with missing data ranging from 1–18%. The point prevalence of flexural dermatitis by clinical exam ranged from 5% to 8%, with a cumulative prevalence of 17% by age 13 and missing data ranging from 1–15% ( Figure 2, Table 1, Supplemental Table 3 ^6^).

*Atopic dermatitis:* The annual maternal or self-reported period prevalence of atopic dermatitis based on the 2-report definition ranged from 13–21% across 12 timepoints, with a cumulative prevalence of 41% by age 18 ( Figure 1). The severity for participants with definite AD ranged from 7–12% for no problem, 51–68% for mild, 18–31% for moderate, and 3–9% for severe AD. The percentage of missing data for period prevalence and severity for each timepoint ranged from 1–2% ( Table 1, Supplemental Table 3 ^6^).

*Other variables related to dermatitis/eczema:* The number of school days taken off due to eczema in the past year ranged from 0.02 (SD 0.3) to 0.03 (0.5) across 3 timepoints. The number of non-missing responses ranged from 3,643 at 11 years to 4,468 at 13 years. The frequency of sleep disturbance due to an itchy rash across 6 timepoints ranged from 93.1–95.3% for never in past 12 months, 3.9–5.7% for less than 1 night per week, to 0.8–1.4% for 1 night or more per week among all children. The number of non-missing responses ranged from 7,600 at age 8 years to 4,797 at age 16 years. Across 3 timepoints from age 10 to 13 years, 10.5% to 13.2% of caregivers reported their children taking medications for eczema in the past year. The number of non-missing responses ranged from 7,097 to 6,253. Out of the total non-missing responses for frequency of medications taken during the same timepoints (7,097 at age 10 years to 6,456 at age 13 years), 1.9–8.5% reported eczema medicine, but no frequency, 28.5–29.6% for regularly, 25.1–27.3% for few days, 27.9–32.6% for odd occasions, and 8.3–9.1% for once or twice in the past year (Supplemental Table 2). ^6^

Participants who reported history of infection with flexural dermatitis in the past year ranged from 3% to 6% across 10 timepoints. The number of non-missing responses ranged from 10,161 at age 2 years to 4,798 at age 16 years. Clearance of flexural dermatitis rash ranged from 76% to 84% across 5 timepoints. The number of non-missing responses ranged from 1,369 to 603 (Supplemental Table 2). ^6^

### Further considerations

*Missing data:* One of the key benefits of the ALSPAC dataset is the repeated measures of dermatitis, eczema, and rash at multiple timepoints. This allows researchers to study longitudinal trajectories and patterns of disease activity and severity, ^10^ which is especially crucial for AD as it is a chronic disease that waxes and wanes over time. ^11^ The longitudinal measures also help reduce the likelihood of reverse causality. Although the study is limited by attrition over time, like other large longitudinal birth cohorts, the repeated measures allow researchers to address sample size issues by combining data from timepoints with and without missing data. ^1^

*Validity of participant report:* Researchers should acknowledge potential misclassification biases, however systematic differential measurement error is unlikely because similar questions are asked at multiple time points. Moreover, the point prevalence estimates from maternal/self-reported AD and from clinical exam are similar (as shown in Figure 2). Additionally, the annual maternal or self-reported period prevalence of atopic dermatitis based on our 2-report definition is similar to the population-based Millennium Cohort Study, which found a lifetime prevalence of 35% by age 5. ^12^

## Conclusions

With a large sample size, longitudinal design, and the availability of data on potential confounding factors that can be linked to the data described in this data note, ALSPAC is a useful data source for studying atopic dermatitis natural history and risk factors and impacts. Extensive data collection on self-reported and clinical data on eczema, atopic dermatitis, and rash allows for research on a wide variety of topics, although missing data and attrition should be considered by researchers as they use this data, as with any other longitudinal studies. Because these measures are asked in different ways and in varying waves of the study, researchers should recognize that their results and conclusions may be affected by the use of different definitions. Our proposed AD definition is most consistent with the ISAAC questionnaire, uses measures that were repeated at the most time points, and produced estimates that align with previous studies.

### Ethics and consent

Ethical approval for the ALSPAC study was obtained from the ALSPAC Ethics and Law Committee (ALEC; IRB00003312) and the Local Research Ethics Committees. Informed consent for the use of data collected via questionnaires and clinics was assumed from participants following the recommendations of the ALSPAC Ethics and Law Committee at the time. The completion of a questionnaire, either on paper or online, was considered to be written consent from participants to use their data for research purposes. For the majority of tests undertaken during face-to-face visits, verbal consent was obtained from participants (both parents and children as appropriate) prior to the start of any data collection. However, some tests required the completion of a written consent form. Full details of the approvals obtained are available from the study website ( http://www.bristol.ac.uk/alspac/researchers/research-ethics/).

## Data Availability

Data were accessed through the Avon Longitudinal Study of Parents and Children ( https://www.bristol.ac.uk/alspac/researchers/access/) and are protected from direct sharing through a data access agreement. Analytic code is available on request from the corresponding author. Access to ALSPAC data is available through a system of managed open access. Application steps to request access to ALSPAC data are highlighted below.
1. Please read the ALSPAC access policy (PDF, 621kB) which describes the process of accessing the data and samples in detail, and outlines the costs associated with doing so. 2. You may also find it useful to browse our fully searchable research proposals database, which lists all research projects that have been approved since April 2011. 3. Please submit your research proposal for consideration by the ALSPAC Executive Committee. You will receive a response within 10 working days to advise you whether your proposal has been approved. Any questions regarding data or sample access should be directed to alspac-data@bristol.ac.uk (data) or bbl-info@bristol.ac.uk (samples). Open Science Framework (OSF). Data Note: Defining prevalence of atopic eczema in the Avon Longitudinal Study of Parents and Children (ALSPAC) Extended Data. https://doi.org/10.17605/OSF.IO/TZEXF. ^6^ This project contains the following underlying data:
• Supplement. A word document containing three supplementary tables. Data is available under the terms of the Creative Commons Attribution 4.0 International license (CC-BY 4.0).
