# Supplementary material for: Defining prevalence of atopic dermatitis in the Avon Longitudinal Study of Parents and Children (ALSPAC)
Source: Wellcome Open Res. 2026 Jul 28;11:249. Originally published 2026 Apr 30. [Version 2] doi: 10.12688/wellcomeopenres.26317.2 (PMC13560793; doi:10.12688/wellcomeopenres.26317.2)
Supplement: Peer review history

# Reviewer report: Reviewer response for version 2

**DOI:** 10.21956/wellcomeopenres.30112.r164218

**Published:** 2026-08-27

**Authors:** Holly Anderton

**Affiliations:** 1 The University of Melbourne Faculty of Medicine Dentistry and Health Sciences (Ringgold ID: 85084), Melbourne, Victoria, Australia

This is a valuable contribution to the literature on atopic dermatitis (AD). Much of the longitudinal literature on AD is derived from patients recruited through dermatology or other clinical settings, which inevitably enriches the study population for moderate-to-severe or otherwise treatment-seeking disease. In contrast, ALSPAC, a population-based birth cohort, provides an opportunity to describe the spectrum of AD at the population level, including the substantial proportion with mild disease. This makes the dataset particularly valuable for understanding the population burden of AD rather than only the experience of clinically selected patients. The authors provide a detailed description of repeated measures of dermatitis, eczema and related symptoms in the ALSPAC cohort from infancy through age 18 and use these data to propose a consistent definition of AD in this cohort based on at least two reports of flexural dermatitis. The resulting definition gives annual prevalence estimates of approximately 13–21%, with a cumulative prevalence of 41% by age 18, and also permits characterisation of disease severity and activity over time.

 The rationale for describing and harmonising the available AD measures within this large longitudinal birth cohort is clear and well justified. The protocols and proposed approach are appropriate, and the manuscript provides sufficient detail regarding the questionnaire items, timing of assessments, clinical examinations, and derivation of the proposed AD categories to allow the methods to be reproduced. The available variables and prevalence estimates are presented clearly and in a usable manner, and the process for obtaining access to the underlying ALSPAC data is adequately described.

 A reassuring feature is the close agreement between the point-prevalence estimates based on maternally/self-reported current flexural dermatitis and those obtained by standardised clinical examination. This concordance provides a useful internal validity check for the questionnaire-based flexural dermatitis measure and supports its usefulness within the proposed definition. The clinical examination data from the Child in Focus substudy provide an interesting opportunity for further internal validation of the proposed AD definition. As currently presented, the prevalence of clinically observed eczema and the mean number of eczema patches at ages 4 and 5 are reported for the examined population as a whole. It would be informative, if feasible, to also report the prevalence of clinically observed eczema and patch prevalence among participants across the four-category AD definitions.

 Overall, I consider this an important and useful data resource that should facilitate future studies of AD incidence, severity, risk factors and outcomes.

Are sufficient details of methods and materials provided to allow replication by others?

Yes

Is the rationale for creating the dataset(s) clearly described?

Yes

Are the datasets clearly presented in a useable and accessible format?

Yes

Are the protocols appropriate and is the work technically sound?

Yes

Reviewer Expertise:

Skin biology, regulated cell death, inflammatory skin disease

I confirm that I have read this submission and believe that I have an appropriate level of expertise to confirm that it is of an acceptable scientific standard.

# Reviewer report: Reviewer response for version 1

**DOI:** 10.21956/wellcomeopenres.28982.r155971

**Published:** 2026-06-15

**Authors:** Allison R Loiselle

**Affiliations:** 1 National Eczema Association, Novato, California, USA

Studying AD over time is complicated by the condition's episodic nature and the lack of standardized terminology across studies. The authors describe measures of dermatitis-related symptoms across time points in the Avon Longitudinal Study of Parents and Children (ALSPAC), a UK birth cohort followed from birth through age 18. Calculated point and period prevalences varied substantially depending on how the condition was defined, underscoring how much case definitions drive prevalence estimates in AD research. To address this, the authors propose a definition of "active" AD, requiring at least two reports of flexural dermatitis, up to and including the time point being considered. Prior research at the University of Bristol nearer the onset of ALSPAC used a similar definition, but only in those 6-42 months in the cohort, while the current definition includes children up to 18 and also adds definitions for inactive and indeterminate AD.

 The study highlights the value of longitudinal, population-based data for understanding AD natural history and the importance of consistent, clearly defined case definitions.

 Based on reviewer guidelines:

 1. The work is clearly and accurately presented and engages with current literature. The authors may add the citation for the ALSPAC team's original article in creating the definition for prevalence of AD (DOI: 10.1111/j.1365-2133.2003.05605.x) (Ref 1) Two small additional comments: there is a misspelling in figure 2 ("peiod" instead of "period") and the order of (“no problem, mild, very bad, or quite bad") in paragraph 5 of the methods should be swapped to match the scale quite -> very as in the tables. 

 2. The study design is appropriate, and the work has academic merit. The concordance between point prevalence estimates from maternal/self-report and clinical exam is a meaningful internal check. Using the ISAAC questionnaire as the anchor for the proposed definition is methodologically sound, given its widespread use and validation in epidemiological studies.

 3. There are sufficient details of methods and analysis provided to allow replication by others. The paper provides a clear and detailed account of the questionnaire items themselves, including the exact wording of the flexural dermatitis question that anchors their proposed AD definition. The timepoints of data collection are well-specified, the distinction between maternal-report and self-report is clearly delineated. The four-category AD classification scheme (never, inactive, maybe, definite) is logically defined and transparently linked to the underlying variables.

 4. The source data underlying the results are not available for direct sharing through a data access agreement.

 5. The conclusions drawn are adequately supported by the results and the authors appropriately mention attrition as a limitation as well as the difference in how questions/measures are asked across different time points in the study.

Are sufficient details of methods and materials provided to allow replication by others?

Yes

Is the rationale for creating the dataset(s) clearly described?

Yes

Are the datasets clearly presented in a useable and accessible format?

Yes

Are the protocols appropriate and is the work technically sound?

Yes

Reviewer Expertise:

Patient-centered research, eczema burden, epidemiology/public health

I confirm that I have read this submission and believe that I have an appropriate level of expertise to confirm that it is of an acceptable scientific standard.

## References

[1] : A prospective study of the prevalence and incidence of atopic dermatitis in children aged 0-42 months. *British Journal of Dermatology* .2003;149(5) : 10.1111/j.1365-2133.2003.05605.x1023-1028 10.1111/j.1365-2133.2003.05605.x 14632809
